# Justification of Discrimination against People with Mental Illness in Republic of Korea

**DOI:** 10.3390/healthcare11081195

**Published:** 2023-04-21

**Authors:** Mikyung Seo, Minhwa Lee, Jinhyang Lee

**Affiliations:** 1 Department of Social Welfare, Gyeongsang National University, Jinju 52828, Republic of Korea; welseo@gnu.ac.kr; 2 Department of Social Welfare, Mokpo National University, Muan 58554, Republic of Korea; 3 Department of Social Welfare, Changshin University, Changwon 51352, Republic of Korea; jinhyang20606@gmail.com

**Keywords:** justification, social distance, Protestant ethic values, morality, mental illness

## Abstract

Aims: This study aimed to analyze the process in which individual values and beliefs affected social distance against people with mental illness by mediating cognition, based on applying the justification–suppression model to the stigma of mental illness. Methods: An online survey was conducted with 491 adults aged 20 to 64 years. Their sociodemographic characteristics, personal values, and beliefs, justification for discrimination, and social distance were measured to assess their perceptions of, and behaviors towards, persons with mental illness. Path analysis was performed to examine the magnitude and significance of the hypothetical relationship between variables. Results: Protestant ethic values and morality significantly affected the justification of inability and dangerousness and attribute responsibility. Excluding attribute responsibility, the justification of inability and dangerousness significantly predicted social distance. In other words, the higher the Protestant ethic values, the higher the morality of binding, and the lower the morality of individualizing, the higher the level of justification based on inability and dangerousness. Such justification has been found to increase social distance from persons with mental illness. In addition, mediating effects were the largest in the path of the morality of binding → justification of dangerousness → social distance. Conclusions: The study proposes various strategies to deal with individual values, beliefs, and justification logic to reduce social distance against those with mental illness. These strategies include a cognitive approach and empathy, both of which inhibit prejudice.

## 1. Introduction

Social stigma refers to the process through which people devalue and exclude certain identities. This process includes stereotypes as concepts learned in the process of socialization, prejudice as affective and cognitive responses to the stereotypes, and discrimination as behavioral responses based on the prejudice [1]. Discrimination is mainly manifested as social distance (SD), which involves avoiding people with specific identities and ignoring their achievements. Therefore, SD can be considered a behavioral indicator of discrimination [2].

Mental illness, a social identity stigmatized in any society, is associated with psychological and behavioral symptoms. However, it is also a social handicap in which the course of the disease is severely affected by cultural and social factors, such as stigma [3]. Due to the importance of social factors in mental illness, studies on social stigma have been conducted consistently from the 1950s until more recent times [2,4,5,6,7,8,9,10,11,12,13,14,15,16]. These studies have shown that there has been a gradual increase in the number of people globally who understand the scientific causes of mental illness and recognize the importance of social integration and support for the recovery of those with mental illness. However, people with mental illness are still perceived as unpredictable and dangerous, with persistent discriminatory behaviors against them, such as avoiding social relationships. This discriminatory reaction of the public limits the various social opportunities essential for recovery, isolating persons with mental illness from society and distancing them from the treatment system. Discrimination worsens mental illness symptoms and increases chronicization and recurrence, thereby further reinforcing the public’s negative reaction [3].

However, through the course of education and in the process of socialization, most people learn that it is wrong to discriminate against any identity, including mental illness. Based on learned social norms, people agree that all humans are equal and that it is unjust to devalue anyone [17]. However, negative perceptions and evaluations of people with mental illness are also learned simultaneously. Therefore, there must be logic to justify unjust behavior, such as discrimination against persons with mental illness. In other words, there should be an explanation for why such discriminatory behavior is acceptable and even desirable, i.e., a justification for such behavior. Crandall and Eshleman [18] proposed the justification–suppression model (JSM) to explain the mechanisms through which prejudice can be expressed. The basic premise of the JSM is that there is a tension between the desire to express prejudice and the desire to maintain one’s values that are contrary to prejudice [19]. In the JSM, suppression is an external or internal motivation for not expressing prejudice, whereas justification is a cognitive framework for expressing prejudice without internal or external sanction [18].

This study focused on how the public justified unjust discrimination against persons with mental illness. Based on the JSM, the only difference between “rational” treatment and “unjust” treatment of the stigmatized is the presence of the justification ideology, which is of two kinds [17]. The first kind consists of “attributional” approaches, in which the focus of justification is on the utilization of the attribution of causality, responsibility, and blame. The second kind consists of “hierarchical” approaches, in which the focus is on the acceptance of hierarchical relations as natural and necessary. Applying this to mental illness, first, discrimination against persons with mental illness is justified by attributing responsibility for mental illness to the subject based on the attributional approach. Second, according to the hierarchical approach, persons with mental illness are deemed inferior, and discrimination against them is considered justified due to their inability. In addition, among the perceptions of the public concerning persons with mental illness, one perception that does not change over time is the perception of dangerousness followed by fear [9,20,21]. The JSM also considered a perceived threat as one of the factors justifying prejudice [18,22]. Therefore, inability, dangerousness, and attribute responsibility (AR) can be listed as justification logic for discriminating against persons with mental illness. Discrimination against those with mental illness is justified as they are dangerous, lack ability, and are responsible for their illness.

In the JSM, Protestant ethic (PE), political orientations, social dominance, etc., were considered individual beliefs or values that justified the expression of prejudice [18]. Among these, PE values claimed that success came only to those who worked hard and denied themselves pleasure. PE is a core value that determines bias against being incompetent and posing harm to the safety of others by considering placing comfort of one’s self and family over a target population and being lazy as sinful [23]. Along with PE, morality is another core value that justifies prejudice against the target group. Morality means the prescriptive judgment of justice and rights [24]. Morality is divided into individualizing morality (individualizing value (IVM)) in which the individual is the locus of moral value and binding morality (binding value (BVM)), in which relations or solidarity are the locus of moral value [24]. In other words, increased endorsement of BVM predicts increased blame and responsibility attributed to the target group; for IVM, it is the opposite. Therefore, in this study, PE and morality (IVM and BVM) were set as values and beliefs that affected the justification logic based on the inability, dangerousness, and AR of people with mental illness.

Based on the JSM, this study examined the process through which PE and morality influenced SD meditated by the justification of inability, dangerousness, and AR of people with mental illness. In the JSM, PE, and morality were regarded as two of the several categories of justification; however, in this study, it was assumed that individual values and beliefs influenced the logic that served to justify one’s discriminatory behavior toward people with mental illness. In other words, it was assumed that values and beliefs influenced cognition and judgment, which in turn determined behavior. Therefore, this study’s research question was as follows: To what extent can individual PE and morality (IVM and BVM) predict SD, a behavioral indicator of discrimination, through the three justification logics—inability, dangerousness, and AR—of people with mental illness? In other words, the study intended to analyze the nine paths that the three personal values took on SD mediated by the three justification logics.

## 2. Materials and Methods

### 2.1. Procedure and Participants

This study was authorized by the Gyeongsang National University’s Institutional Review Board (IRB). Through Macromill Embrain, a company with a platform for online surveys, the study was accessible to the public from February to March 2022. Online surveys begin with eligibility questions (age, gender, and region) by the population distribution in Republic of Korea and informed consent as approved by the study IRB. There was a total of 491 participants who responded anonymously, and no identifying data were gathered. Informed consent to take part in the survey was obtained from all participants.

Table 1 presents the participants’ sociodemographic characteristics. Regarding gender, there were 247 males (50.3%) and 244 females (49.7%). Participants’ average age was 46.73 ± 10.64 (years), with 45 participants aged 20 to 29 (9.2%), 64 aged 30 to 39 (13.0%), 148 aged 40 to 49 (30.1%), 171 aged 50 to 59 (34.8%), and 63 aged 60 to 64 (12.8%) years. The average number of years of education was 18.25 ± 1.73 (years), with 81 participants having a high school degree (16.5%), 68 having a college degree (13.8%), 296 having a university degree (60.3%), and 46 having a graduate school degree (9.4%). Participants’ average monthly income was 369.76 ± 258.73 (KRW).

### 2.2. Measure

#### 2.2.1. Protestant Ethic

PE was defined as the dedication to the values of hard effort, to work as an end itself, and to the workplace as the preferred structure for fulfilling internalized values [25]. The PE scale developed and validated by Mirels and Garrett [26] was used to measure PE in this study. The original scale consisted of 19 items on a seven-point scale with scores from −3 to 3. On the original scale, this study used 17 questions excluding the following 2 items which judged a similar meaning to other items: “people ought to have more free time to spend on rest” and “any individual who can and is ready to work hard has a good chance of success.” A higher score corresponded to a greater PE value. Cronbach’s alpha was 0.761.

#### 2.2.2. Morality

Morality was described as “prescriptive judgments of rights, justice, etc., about how individuals should interact with one other” [27]. To measure morality, this study used 20 moral judgment items reported by Graham et al. [28]. This study’s scale was a six-point scale consisting of IVM and BVM factors. A higher IVM score was associated with the tendency of helping those in need, refraining from harming others, and valuing fairness, while a higher BVM score was associated with the tendency of being loyal to the group, respecting authority, and emphasizing self-control [28]. Cronbach’s alpha was 0.747 for IVM and 0.660 for BVM.

#### 2.2.3. Justification of Discrimination

The justification is a cognitive framework for expressing prejudice without internal or external sanction [18]. In this study, the justification of discrimination against persons with mental illness was measured from three aspects: inability, dangerousness, and AR. The scale developed by Park and Seo [29] was used to measure justification based on inability and dangerousness, i.e., the recognition that it can be considered justifiable to restrict the liberty of persons with mental illness for efficiency, social safety, and positive outcomes. This scale was a five-point scale consisting of nine items on the inability (JI) and six items on the dangerousness (JD). A higher score indicated a higher perception that discrimination based on inability or dangerousness was justified. For AR, the four items on the responsibility factor of the attribution scale used by Mantler et al. [30] were used. Responsibility was measured with scales that consisted of two positively and two negatively worded statements. Each item was scored using a five-point scale, a higher score meant a greater level of perception that people with mental illness were responsible for their disease. Cronbach’s alpha was 0.907 for JI, 0.852 for JD, and 0.627 for AR.

#### 2.2.4. Social Distance

SD refers to the relative willingness to participate in some relationships or the degree desired to distance oneself from others and was commonly considered a public attitude toward persons with mental illness [2]. SD was measured using the discrimination scale developed by the NHRC of Korea [31]. It asked about their intention to interact with those with mental illness as friends, neighbors, or coworkers and to offer chances to lend to or hire them. Each item was scored from 1 to 5, and a higher score was associated with a higher SD for people with mental illness. Cronbach’s alpha was 0.914.

### 2.3. Data Analysis

The data were analyzed using SPSS 27.0 and Amos 27.0 for Windows (IBM Corp., Armonk, NY, USA) program. Before analysis, the normality of the main variables was confirmed, and the goodness-of-fit of the measurement model was evaluated through confirmatory factor analysis (CFA). Cronbach’s alpha was used to verify the scale’s reliability. First, frequency and descriptive statistics were used to examine the sociodemographic features of the participants. Second, differences in the variables according to the features of the participants were analyzed by conducting independent samples t-test and using Pearson’s correlation. Third, the multicollinearity between the independent variables was analyzed using Pearson’s correlation and regression analysis. Fourth, path analysis was conducted to examine the validity of the causal relationship between the variables and the direct and indirect effects between them [32,33]. That is, it was utilized to test the hypothesis that a model of independent, mediating, and dependent variables congruent with the justification–suppression model demonstrate adequate fit to the data collected in this cross-sectional study.

## 3. Results

### 3.1. Characteristics of the Variables

Table 2 shows descriptive statistics of the variables. The mean of the variables was 4.21 (±0.69) for PE, 4.94 (±0.62) for IVM, and 4.04 (±0.52) for BVM. As for the three types of justification for discrimination, JI, JD, and AR, the mean was 2.67 (±0.69), 2.79 (±0.70), and 2.88 (±0.67), respectively, and 3.18 (±0.76) for SD.

### 3.2. Differences in the Variables According to the Sociodemographic Variables

Table 3 shows the average differences in the variables based on sociodemographic characteristics. First, regarding the difference in the variables according to gender, PE was higher in males (4.30 ± 0.68) than in females (4.11 ± 0.70), whereas SD was higher in females (3.29 ± 0.76) than in males (3.07 ± 0.75). Other variables were at a similar level, and there was no statistically significant difference. Examining the relationship between age and the variables revealed that all variables except AR showed a statistically significant positive correlation with age. In other words, the higher the age, the greater the levels of PE (r = 0.229), IVM (r = 0.229), BVM (r = 0.193), JI (r = 0.170), JD (r = 0.219), and SD (r = 0.144). Examining the relationship between monthly average income and the variables revealed that only PE showed a meaningful positive correlation. In other words, the higher the monthly average income, the greater the PE (r = 0.108) level. However, there was no significant relationship between education level and the other variables.

### 3.3. Results of the Path Analysis

#### 3.3.1. Evaluation of the Goodness-of-Fit for the Path Model

Through confirmatory factor analysis (CFA), the goodness-of-fit indices of the full measurement model were the chi-square = 416.604 (*p* = 0.000), CMIN/DF = 3.205, SRMR = 0.0812, GFI = 0.913, AGFI = 0.873, NFI = 0.918, RFI = 0.892, IFI = 0.942, TLI = 0.923, CFI = 0.941, RMSEA = 0.067. Most of the indices satisfied the corresponding acceptance criteria.

Table 4 presents the results obtained by evaluating the goodness-of-fit for the path model. This model was proven to be based on values of the Absolute Fit Index, the Incremental Fit Index, and the standardized root means square residual (SRMR) which was far less than 0.08. In summary, the fit indices indicate that hypothesized model is a good fit to the collected data.

#### 3.3.2. Direct and Indirect Effect of Variables on SD

Table 5 shows the direct and indirect effects of the path model. First, the direct effect of PE on the three types of justification was examined. All paths from PE to JI (B = 0.228), JD (B = 0.124), and AR (B = 0.113) had a positive direct effect, which was statistically significant. All paths from IVM to JI (B = −0.272), JD (B = −0.227), and AR (B = −0.196) had a negative direct effect, which was also statistically significant. Finally, all paths from BVM to JI (B = 0.368), JD (B = 0.451), and AR (B = 0.260) had a positive direct effect, which was also statistically significant. Therefore, the direct effects of the three predicting variables on the three mediating variables were found to be statistically significant. In other words, with a high level of PE, a low level of IVM, and a high level of BVM, the level of justification based on inability, dangerousness, and AR increased. Second, the direct effects of the three types of justification on SD were examined. While the paths of JI → SD (B = 0.226) and JD → SD (B = 0.569) were statistically significant, the path of AR → SD was not. This meant that the discriminatory responses regarding not having a personal relationship with those with mental illness increased in tandem with the perception that those with mental illness deserved unfair treatment since they lacked ability and were dangerous. However, the AR-based justification that those with mental illness were responsible for their disability did not predict SD significantly. Third, the indirect effects of PE, IVM, and BVM on SD were examined. The analysis revealed that only six of the nine paths in which the three independent variables affected SD mediated by the three justifications were statistically significant. In other words, the paths of PE → JI → SD (B = 0.052), PE → JD → SD (B = 0.071), IVM → JI → SD (B = −0.061), IVM → JD → SD (B = −0.129), BVM → JI → SD (B = 0.083), and BVM → JD → SD (B = 0.257) were significant. Therefore, a high level of PE, a low level of IVM, and a high level of BVM were associated with a high level of justification based on the inability or dangerousness of those with mental illness, and this justification increased SD in people with mental illness. Since the direct effect of AR → SD was not statistically significant, the indirect effect of the three paths mediated by AR was also not significant. Additionally, comparing the paths with the significant indirect effect, the path of BVM → JD → SD best explained the process of justifying SD against people with mental illness.

**Figure 1:**
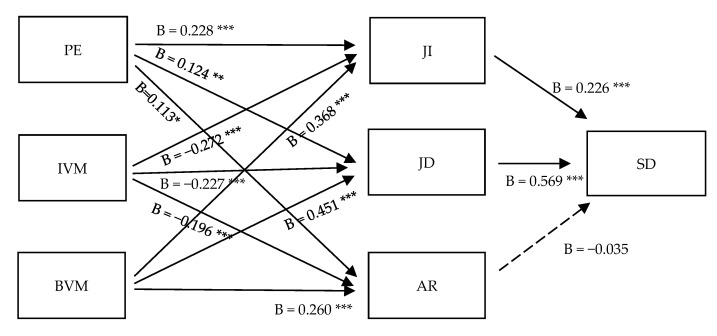
Path model for SD. * *p* < 0.05, ** *p* < 0.01, *** *p* < 0.001. A solid line denotes statistical significance, but dotted line does not.

Figure 1 represents the path coefficient and significance of independent variables (PE, IVM, and BVM) leading to SD mediated by justification (JI, JD, and AR).

## 4. Discussion

This study aimed to analyze the process whereby discrimination against persons with mental illness was justified according to the JSM. To this end, it conducted a survey with 491 Korean adults sampled online, considering the age, region, and gender ratio of the participants. The survey included PE, morality, three justifications based on inability, dangerousness, and AR, and SD, a behavioral indicator of discrimination, concerning people with mental illness. The study revealed several key findings.

First, in the relationship between the sociodemographic characteristics and the main variables, PE was lower and SD was higher in females than in males. This was in agreement with the results from other studies, where women were found to be more prejudiced and discriminatory against people with mental illness, when compared to men [31,34]. Interestingly, PE was higher in males than in females. In terms of the sociodemographic characteristics, the level of PE, BVM, JI, JD, and SD increased with increasing age. This was consistent with the results of other studies that showed that as one gets older, one becomes more conservative [35] and more prejudiced against people with mental illness [16,29,31]. Only PE showed a significant positive correlation with participants’ income. This suggested that people were more likely to be financially successful with a higher level of focus on the values of work and success.

Second, PE and morality (IVM and BVM) significantly predicted JI and JD for people with mental illness. PE is based on beliefs such as “people get what they deserve” or “people who work deserve success.” [18]. BVM is based on the belief that behavior that violates conservative values, such as obedience, discipline, or the preservation of tradition, is considered immoral [24]. Therefore, these two values indicate that persons with mental illness who lack the ability and may be harmful to themselves and others deserve discriminatory treatment toward them. Previous studies [23,24,36] have also presented PE and BVM as the core values that could explain prejudice against sexual minorities, victims of sexual violence, and people of color. Unlike BVM, IVM focuses on promoting impartial care and unconditionally prohibiting harm and is associated with sensitivity to victim suffering. Therefore, IVM reflects liberal characteristics [37,38,39], associated with increasing care and helping the stigmatized [24]. This study confirmed that the influence of these beliefs could also be applied to prejudice against persons with mental illness. Therefore, the level of justification based on dangerousness (B = 0.124, B = 0.451), inability (B = 0.228, B = 0.368), and AR (B = 0.113, B = 0.260) was increased with an increase in the level of PE and BVM. In contrast, the level of justification based on dangerousness (B = −0.227), inability (B = −0.272), and AR (B = −0.196) was significantly decreased with an increasing level of IVM. 

Third, among the three justification logics, JD was the most significant predictor of SD, followed by JI. JD stems from a long-standing prejudice that “persons with mental illness are dangerous.” Prejudice regarding dangerousness has been a basis for paternalists justifying forced hospitalization or restricted freedom for the safety of the majority [29,40,41]. This study’s findings are aligned with these claims. However, there is no objective evidence that persons with mental illness are dangerous, and the crime rate in persons with mental illness has been reported to be lower than in those without mental illness [42,43]. Nevertheless, fear disseminated by media without direct experience tends to aggravate the perception of dangerousness regarding people with mental illness [44,45,46]. JI refers to the logic that “persons with mental illness deserve unfair treatment because they lack ability.” Although the inability of persons with mental illness may be due to their illness, it may also be due to limited opportunities owing to social stigma. In other words, community integration, which must precede the recovery of people with mental illness [47,48,49], is possible only through employment, housing, and participation in various community activities. Therefore, discrimination in these areas limits the opportunities for persons with mental illness to demonstrate their abilities and is a factor that exacerbates their inability. Finally, discrimination reinforces incapacity, which in turn reinforces prejudice regarding inability, and it is difficult to determine which comes first.

Fourth, this study assumed that PE and morality would predict SD mediated by AR based on an attributional approach. However, while both PE and morality significantly predicted AR for illness, AR-based justification did not predict SD significantly. Considering that previous studies [50,51] reported that attributing responsibility to stigmatized targets or victims or blaming them predicted discrimination, this study’s results were rather unexpected. Furthermore, the premise of the attribution theory by Weiner [52], which argues that discrimination increases due to anger when attributing responsibility to the target and considers the cause as controllable, was not consistent with this study’s results. Two interpretations are possible in this case. First, several studies [53,54,55] have argued that the attribution theory required modification for people with mental illness, unlike the case of other stigmas. In other words, when the cause was considered uncontrollable and the target was not held responsible, their behavior would be more difficult to predict. This increased the fear that they may not be able to control their behavior, thereby increasing discrimination. Significantly, these studies showed that fear increased when the cause of the mental disorder was recognized as an uncontrollable biological cause. Therefore, the focus of these studies was somewhat different from that of the present study, which attributed responsibility for the illness. However, their results were in line with that of the present study in that they revealed that the attributional approach may not apply to mental illness. Second, the attribution-value model was highly correlated with discrimination in individualistic cultures; such a correlation has been known to be weakened in collectivist cultures, where the judgment of personal responsibility is not central to perception [18,56]. Significantly, Republic of Korea is a representative country with a collectivist culture, where people value social harmony, respectfulness, and group needs over individual needs. However, to support the interpretation related to culture, further research is required on how the attribution of responsibility for mental illness differs between cultures. 

Fifth, the paths of PE, IVM, and BVM on SD mediated by the two justification logics were statistically significant in six pathways, which excluded AR. This indicated that personal values or beliefs influenced the logic of justifying discrimination based on inability and dangerousness, which in turn predicted SD. In general, morality and ethical beliefs are innate characteristics that do not change easily and work as a framework for recognizing the phenomenon; however, they can be modified through various experiences [24,37]. These values and beliefs learned from childhood contribute to constructing the logic that serves to justify unjust discrimination against persons with mental illness and also against all social minorities who are stigmatized. Therefore, it is necessary to study various experiences in which values and beliefs can be changed.

## 5. Conclusions

Based on the JSM, this study analyzed the effect of individual values, PE, and morality (IVM and BVM), on SD mediated by the three justifications based on JI, JD, and AR. Unlike previous studies on social stigmas associated with mental illness (people with mental illness), this study analyzed the process in which discrimination occurred and how people justified unjust discriminatory behavior against people with mental illness. Several suggestions were made based on the findings, as follows: First, the JSM presented egalitarianism, political liberalism, and internal standards based on the belief that they should be entirely non-prejudiced as suppression sources of expressing prejudice [18]. These values and beliefs must be learned from an early age. They are crucial to pursuing an equal and just society since they support fair treatment for all stigmatized groups, not just people with mental illness. This study also confirmed that IVM focused on equality and minority protection had a negative influence on all three justification logics. Second, justification was logic. In other words, the logic that “people with mental illness lack ability and are dangerous and therefore deserve to be treated accordingly” seemed to affect SD, which was a behavioral indicator. Therefore, a cognitive approach that refutes this justification logic can be proposed. Efforts are needed to confirm that “people with mental illness are no more dangerous than those without illness and that their incapacity can be supplemented through social support and various opportunities” through various objective data. This study proposed a media-based approach. While misinformation transmitted through the media may lead the public to perceive that people with mental illness are dangerous and lack the ability [44,45,46], the media can also be effective in changing this perception. It can be utilized for the widespread dissemination of objective data that refute the misconception that people with mental illness are dangerous. The media can also be utilized to change public perception through documentaries or public service advertisements that introduce people who have succeeded in various fields despite living with a mental illness. This may have already been pointed out in several previous studies [57,58,59,60]. Third, empathy is a meaningful strategy that can suppress the expression of prejudice. According to the JSM, empathy for one person in a group can result in reducing prejudice against the group as a whole [18]. In general, since empathy is defined as the ability to recognize the suffering of others and help them [61], it can predict a positive response even if there is no change in stereotypes. Therefore, various contact experiences are suggested as a strategy to improve empathy [62]. Recently, a method using virtual reality has also been proposed in this regard [63,64].

## 6. Limitations

Unlike previous studies on social stigma against people with mental illness, which analyzed the level of prejudice and discrimination, this study is novel in that it analyzed how discrimination was justified based on the JSM. However, this study has several limitations. First, although the original JSM model explained the meaningful relationship between suppression and justification factors, this study only focused on justification and predicted SD. This means that only a part of the JSM model has been verified. Therefore, a follow-up study should clarify the role of suppression by applying the original JSM model to mental illness. The influence of suppression is particularly important in suggesting a realistic alternative to anti-stigma. Second, considering the limited number of studies on how discrimination against people with mental illness was justified, we had difficulty finding an appropriate justification measure. The scale we used was extracted from the items that justified the violation of the liberty of people with mental illness. Therefore, the influence of various other factors related to justification, such as covering and social roles, could not be considered by applying only the justification logic based on inability, dangerousness, and AR, as revealed in previous studies [17,18]. Third, as numerous studies have shown, contact with mental illness has a significant impact on their social stigma. The contact level, however, was not controlled in this study. Because values and beliefs can influence contact experience, and vice versa, contact experience influences beliefs, more research is needed to account for this. Fourth, South Korea is a country with a collectivist culture. To determine whether AR was statistically insignificant in this study due to South Korea’s cultural differences, a comparison with different cultures is necessary. Fifth, this study has the usual limitations related to self-reported studies and data acquired using cross-sectional methods.

## Figures and Tables

**Table 1:** Sociodemographic characteristics of the participants.

Category	Frequency (N)	Percentage (%)
Total	491	100
Gender		
Male	247	50.3
Female	244	49.7
Age	46.73 ± 10.64	
20–29	45	9.2
30–39	64	13.0
40–49	148	30.1
50–59	171	34.8
60–64	63	12.8
Education	18.25 ± 1.73	
High School	81	16.5
College	68	13.8
University	296	60.3
Graduate School	46	9.4
Income	369.76 ± 258.73	

**Table 2:** Descriptive statistics of the variables.

Variable	Range	Min	Max	M ± SD	Skewness	Kurtosis
PE	1–7	1.92	6.67	4.21 ± 0.69	0.160	0.182
IVM	1–6	3.14	6.00	4.94 ± 0.62	−0.425	−0.323
BVM	1–6	2.36	5.91	4.04 ± 0.52	0.211	0.464
JI	1–5	1.00	5.00	2.67 ± 0.69	−0.146	0.025
JD	1–5	1.00	4.83	2.79 ± 0.70	−0.252	0.083
AR	1–5	1.00	5.00	2.88 ± 0.67	−0.055	0.334
SD	1–5	1.00	5.00	3.18 ± 0.76	−0.216	0.123

**Table 3:** Differences in the variables according to the demographic variables.

Variable	PE	IVM	BVM	JI	JD	AR	SD
Gender							
Male	4.30	4.89	4.08	2.67	2.75	2.93	3.07
	±0.68	±0.65	±0.52	±0.67	±0.67	±0.68	±0.75
Female	4.11	5.00	4.01	2.68	2.83	2.83	3.29
	±0.70	±0.60	±0.52	±0.71	±0.74	±0.66	±0.76
t	3.022 **	−1.930	1.653	−0.294	−1.213	1.629	3.223 **
Age (r)	0.229 **	0.229 **	0.193 **	0.170 **	0.219 **	0.010	0.144 **
Education (r)	−0.046	0.039	−0.051	−0.034	−0.034	0.036	−0.002
Income (r)	0.108 *	−0.042	0.018	0.074	0.063	0.065	0.043

* *p* < 0.05, ** *p* < 0.01

**Table 4:** Goodness-of-fit of the path model.

Goodness-of-Fit Measure	Statistics
Absolute fit index	Chi-Square = 22.106, df = 3, *p* = 0.000
	SRMR = 0.0324, GFI = 0.988
Incremental fit index	NFI = 0.983, IFI = 0.985, TLI = 0.893, CFI = 0.985

**Table 5:** Estimation results of the path model.

Path		Direct Effect			Indirect Effect	95% CI Lower, Upper	Total Effect
		B	β	S.E.			
1.	PE → JI	0.228 ***	0.228	0.046	0.052 **	0.019, 0.104	0.118 **
	JI → SD	0.226 ***	0.206	0.063			
2.	PE → JD	0.124 **	0.122	0.048	0.071 *	0.012, 0.143	
	JD → SD	0.569 ***	0.526	0.062			
3.	PE → AR	0.113 *	0.117	0.048	−0.004	−0.020, 0.004	
	AR → SD	−0.035	−0.031	0.039			
4.	IVM → JI	−0.272 ***	−0.245	0.048	−0.061 **	−0.109, −0.025	−0.184 ***
	JI → SD	0.226 ***	0.206	0.063			
5.	IVM → JD	−0.227 ***	−0.201	0.050	−0.129 ***	−0.196, −0.074	
	JD →SD	0.569 ***	0.526	0.062			
6.	IVM → AR	−0.196 ***	−0.182	0.050	0.007	−0.007, 0.028	
	AR → SD	−0.035	−0.031	0.039			
7.	BVM → JI	0.368 ***	0.277	0.065	0.083 **	0.030, 0.156	0.331 ***
	JI → SD	0.226 ***	0.206	0.063			
8.	BVM → JD	0.451 ***	0.334	0.067	0.257 ***	0.167, 0.361	
	JD → SD	0.569 ***	0.526	0.062			
9.	BVM → AR	0.260 ***	0.202	0.067	−0.009	−0.037, 0.009	
	AR → SD	−0.035	−0.031	0.039			

* *p* < 0.05, ** *p* < 0.01, *** *p* < 0.001

## Data Availability

Restrictions apply to the availability of these data. Data were obtained from the participants and are available with the permission of the participants.

## References

[1] Overton S.L., Medina S.L. (2008). The stigma of mental illness. J. Couns. Dev..

[2] Lauber C., Nordt C., Falcato L., Rössler W. (2004). Factors influencing social distance toward people with mental illness. Community Ment. Health J..

[3] Seo M.K., Lee M.H., Park G.W. (2020). Social Stigma and Mental Disorder.

[4] Ko B.J. (1979). A community survey on attitude toward ex-mental patients. J. Korean Neuropsychiatr. Assoc..

[5] Lee E.H., Kim K.J., Lee S.Y. (2000). The attitudes of the inhabitants of Kwangju towards the persons with mental illness. J. Korean Neuropsychiatr. Assoc..

[6] Kim C.N., Seo M.K. (2004). A study on prejudice and discrimination against the mentally ill. Korean J. Health Psychol..

[7] Park G.W., Seo M.K. (2012). A study of prejudice and discrimination against person with mental illness of college students. Soc. Sci. Res. Rev..

[8] Moon N.Y., Kim S.S., Gil M.J. (2018). Factors associated with discriminatory behavior toward people with mental disorders. Health Soc. Welf. Rev..

[9] National Human Rights Commission of Korea (2019). Survey on the Implementation of the National Report on Mental Disabilities.

[10] Borinstein A.B., Spaniol L.J., Gagne C., Koehler M. (1997). Public attitudes toward persons with mental illness. Psychological and Social Aspects of Psychiatric Disability.

[11] Link B.G., Phelan J.C., Bresnahan M., Stueve A., Pescosolido B.A. (1999). Public conceptions of mental illness: Labels, causes, dangerousness, and social distance. Am. J. Public. Health.

[12] Phelan J.C., Link B.G., Stueve A., Pescosolido B.A. (2000). Public conceptions of mental illness in 1950 and 1996: What is mental illness and is it to be feared?. J. Health Soc. Behav..

[13] Corrigan P.W., Green A., Lundin R., Kubiak M.A., Penn D.L. (2001). Familiarity with and social distance from people who have serious mental illness. Psychiatr. Serv..

[14] Angermeyer M.C., Matschinger H. (2005). Causal beliefs and attitudes to people with schizophrenia: Trend analysis based on data from two population surveys in Germany. Br. J. Psychiatry.

[15] Angermeyer M.C., Matschinger H., Schomerus G. (2013). Attitudes towards psychiatric treatment and people with mental illness: Changes over two decades. Br. J. Psychiatry.

[16] Schomerus G., Van der Auwera S., Matschinger H., Baumeister S.E., Angermeyer M.C. (2015). Do attitudes towards persons with mental illness worsen during the course of life? An age-period-cohort analysis. Acta Psychiatr. Scand..

[17] Crandall C.S., Heatherton T.F., Kleck R.E., Hebl M.R., Hull J.G. (2000). Ideology and lay theories of stigma: The justification of stigmatization. The Social Psychology of Stigma.

[18] Crandall C.S., Eshleman A. (2003). A justification-suppression model of the expression and experience of prejudice. Psychol. Bull..

[19] Bays A. (2018). The Justification of Prejudice toward Childfree Women. Ph.D. Thesis.

[20] Martin J.K., Pescosolido B.A., Tuch S.A. (2000). Of fear and loathing: The role of ‘disturbing behavior’, labels, and causal attributions in shaping public attitudes toward people with mental illness. J. Health Soc. Behav..

[21] Corrigan P.W., Larson J., Sells M., Niessen N., Watson A.C. (2007). Will filmed presentations of education and contact diminish mental illness stigma?. Community Ment. Health J..

[22] Bahns A.J. (2017). Threat as justification of prejudice. Gr. Process. Intergr. Relat..

[23] Biernat M., Vescio T.K., Theno S.A. (1996). Violating American values: A “value congruence” approach to understanding outgroup attitudes. J. Exp. Soc. Psychol..

[24] Graham J., Nosek B.A., Haidt J., Iyer R., Koleva S., Ditto P.H. (2011). Mapping the moral domain. J. Pers. Soc. Psychol..

[25] Boshoff C., Arnolds C. (1995). Some antecedents of employee commitment and their influence on job performance: A multi foci study. South Afr. J. Bus. Manag..

[26] Mirels H.L., Garrett J.B. (1971). The Protestant ethic as a personality variable. J. Consult. Clin. Psychol..

[27] Turiel E. (1983). The Development of Social Knowledge: Morality and Convention.

[28] Graham J., Haidt J., Nosek B.A. (2009). Liberals and conservatives rely on different sets of moral foundations. J. Pers. Soc. Psychol..

[29] Park G.W., Seo M.K. (2020). The public’s justification for the rights guarantee and infringement of people with mental illness. J. Soc. Sci..

[30] Mantler J., Schellenberg E.G., Page J.S. (2003). Attributions for serious illness: Are controllability, responsibility and blame different constructs?. Can. J. Behav. Sci..

[31] National Human Rights Commission of Korea (2008). A Survey on Discrimination and Prejudice against People with Mental Illness.

[32] Bae B.R. (2017). Structural Equation Modeling with Amos 24.

[33] Streiner D.L. (2005). Finding our way: An introduction to path analysis. Can. J. Psychiatry.

[34] Park J.H., Lee H.S. (2019). A study on the use of the media for positive perception and attitude towards people with mental illness: Focusing on depression, obsessive-compulsive disorder, and panic disorder. Korea Acad. Coop. Soc..

[35] Choi P.S., Min I.S. (2015). The effects of the elderly voters on conservative political attitudes: Focusing on recent nationwide elections. Surv. Res..

[36] Niemi L., Young L. (2016). When and Why we see victims as responsible: The impact of ideology on attitudes toward victims. Pers. Soc. Psychol. Bull..

[37] Kim K.R., Kang J.S., Yun S.Y. (2012). Moral intuitions and political orientation: Similarities and differences between South Korea and the United States. Psychol. Rep..

[38] Seok S.H., Jang Y.B., Ryu S.H. (2015). Are Korean moderates amoral?: Comparison on moral foundations among different political orientation groups. Korean J. Sociol..

[39] Lee J.H., Cho G.H. (2014). Differences in moral foundations between liberals and conservatives. Korean J. Soc. Pers. Psychol..

[40] Carpenter J. (2006). Predictors of Experienced Coercion among Mental Health Service Recipients. Ph.D. Thesis.

[41] Priebe S., Katsakou C., Amos T., Leese M., Morriss R., Rose D., Wykes T., Yeeles K. (2009). Patients’ views and readmissions 1 year after involuntary hospitalisation. Br. J. Psychiatry.

[42] Hwang S.D. (1993). A study on the relationship between mental illness and crime—Comparative analysis of crime between mentally ill people and the general public. Korean J. Soc. Welf..

[43] Korean National Police Agency (2014). Police Statistical Yearbook 2013.

[44] Wahl O.F. (1992). Mass media images of mental illness: A review of the literature. J. Community Psychol..

[45] Smith B. (2015). Mental illness stigma in the media. Rev. J. Undergrad. Stud. Res..

[46] Park S.J., Shin N.R., Kim S.H., Park S.B., Kim C.E. (2020). Semantic network analysis of issues related to mental illness in Korea media: Focusing on the five major media from 2016 to 2018. J. Korean Neuropsychiatr. Assoc..

[47] Bond G.R., Salyers M.P., Rollins A.L., Rapp C.A., Zipple A.M. (2004). How evidence-based practices contribute to community integration. Community Ment. Health J..

[48] Yanos P.T., Barrow S.M., Tsemberis S. (2004). Community integration in the early phase of housing among homeless persons diagnosed with severe mental illness: Successes and challenges. Community Ment. Health J..

[49] Lee M.H., Seo M.K. (2020). Community integration of persons with mental disorders compared with the general population. Int. J. Environ. Res. Public Health.

[50] Watson A.C. (2002). Mental Illness Stigma: Ideology, Causal Attributions, Perceptions of Dangerousness, and Behavioral Response. Ph.D. Thesis.

[51] Corrigan P.W., Markowitz F.E., Watson A.C., Rowan D., Kubiak M.A. (2003). An attribution model of public discrimination towards persons with mental illness. J. Health Soc. Behav..

[52] Weiner B. (1995). Judgments of Responsibility: A Foundation for a Theory of Social Conduct.

[53] Park K.W., Seo M.K. (2012). A modified attribution-affection model of public discrimination against persons with mental illness-model comparisons among schizophrenia, depression and alcoholism. Korean J. Soc. Welf..

[54] Read J., Haslam N., Sayce L., Davies E. (2006). Prejudice and schizophrenia: A review of the ‘mental illness is an illness like any other’ approach. Acta Psychiatr. Scand..

[55] Dietrich S., Matschinger H., Angermeyer M.C. (2006). The relationship between biogenetic causal explanations and social distance toward people with mental disorders: Results from a population survey in Germany. Int. J. Soc. Psychiatry.

[56] Hegarty P., Golden A.M. (2008). Attributional beliefs about the controllability of stigmatized traits: Antecedents or justifications of prejudice?. J. Appl. Soc. Psychol..

[57] Clement S., Van Nieuwenhuizen A., Kassam A., Flach C., Lazarus A., De Castro M., McCrone P., Norman I., Thornicroft G. (2012). Filmed v. live social contact interventions to reduce stigma: Randomised controlled trial. Br. J. Psychiatry.

[58] Penn D.L., Chamberlin C., Mueser K.T. (2003). The effects of a documentary film about schizophrenia on psychiatric stigma. Schizophr. Bull..

[59] Vila-Badia R., Martínez-Zambrano F., Arenas O., Casas-Anguera E., García-Morales E., Villellas R., Martín J.R., Pérez-Franco M.B., Valduciel T., Casellas D. (2016). Effectiveness of an intervention for reducing social stigma towards mental illness in adolescents. World J. Psychiatry.

[60] Thonon B., Pletinx A., Grandjean A., Billieux J., Larøi F. (2016). The effects of a documentary film about schizophrenia on cognitive, affective and behavioural aspects of stigmatisation. J. Behav. Ther. Exp. Psychiatry.

[61] Lee M.H., Seo M.K. (2021). The empathy and working relationships of public sector case managers with people with mental illness. Health Soc. Study.

[62] Soral W., Malinowska K., Bilewicz M. (2022). The role of empathy in reducing hate speech proliferation. Two contact-based interventions in online and off-line settings. Peace Confl. J. Peace Psychol..

[63] Tassinari M., Aulbach M.B., Jasinskaja-Lahti I. (2022). Investigating the influence of intergroup contact in virtual reality on empathy: An exploratory study using AltspaceVR. Front. Psychol..

[64] Bujić M., Salminen M., Macey J., Hamari J. (2020). “Empathy machine”: How virtual reality affects human rights attitudes. Internet Res..

